# Differential Expression of Non-Shelterin Genes Associated with High Telomerase Levels and Telomere Shortening in Plasma Cell Disorders

**DOI:** 10.1371/journal.pone.0137972

**Published:** 2015-09-14

**Authors:** Julieta Panero, Flavia Stella, Natalia Schutz, Dorotea Beatriz Fantl, Irma Slavutsky

**Affiliations:** 1 Laboratorio de Genética de Neoplasias Linfoides, Instituto de Medicina Experimental, CONICET-Academia Nacional de Medicina, Buenos Aires, Argentina; 2 Departamento de Clínica Médica, Sección Hematología, Hospital Italiano de Buenos Aires, Buenos Aires, Argentina; University of Newcastle, UNITED KINGDOM

## Abstract

Telomerase, shelterin proteins and various interacting factors, named non-shelterin proteins, are involved in the regulation of telomere length (TL). Altered expression of any of these telomere-associated genes can lead to telomere dysfunction, causing genomic instability and disease development. In this study, we investigated the expression profile of a set of non-shelterin genes involved in essential processes such as replication (*RPA1*), DNA damage repair pathways (MRE11-RAD50-NBS1) and stabilization of telomerase complex (*DKC1*), in 35 patients with monoclonal gammopathy of undetermined significance (MGUS) and 40 cases with multiple myeloma (MM). Results were correlated with *hTERT* expression, TL and clinical parameters. Overall, a significant increase in *DKC1*, *RAD50*, *MRE11*, *NBS1* and *RPA1* expression along with an upregulation of *hTERT* in MM compared with MGUS was observed (p≤0.032). Interestingly, in both entities high mRNA levels of non-shelterin genes were associated with short TLs and increased *hTERT* expression. Significant differences were observed for *DKC1* in MM (p ≤0.026), suggesting an important role for this gene in the maintenance of short telomeres by telomerase in myeloma plasma cells. With regard to clinical associations, we observed a significant increase in *DKC1*, *RAD50*, *MRE11* and *RPA1* expression in MM cases with high bone marrow infiltration (p≤0.03) and a tendency towards cases with advanced ISS stage, providing the first evidence of non-shelterin genes associated to risk factors in MM. Taken together, our findings bring new insights into the intricate mechanisms by which telomere-associated proteins collaborate in the maintenance of plasma cells immortalization and suggest a role for the upregulation of these genes in the progression of the disease.

## Introduction

Multiple myeloma (MM) is a malignant B-cell lymphoproliferative disease, postgerminal, characterized by the infiltration of clonal plasma cells in the bone marrow (BM) that secretes a monoclonal protein in the majority of patients [1]. It accounts for 10% of all hematologic malignancies and represents the second most common hematologic cancer [2]. At the genetic level, MM is a heterogeneous disease characterized by multistage accumulation of genetic abnormalities deregulating different pathways. MM develops from a premalignant condition, monoclonal gammopathy of undetermined significance (MGUS), often through an intermediate stage termed smoldering multiple myeloma, which differs from active myeloma by the absence of disease-related end-organ damage. MGUS is characterized by plasma cell content of less than 10% in the BM and M-protein in serum < 30 g/L, and no hypercalcemia, renal failure, anemia, and bone lesions (referred to as CRAB features) [3]. As the molecular bases underlying such malignant evolution have not yet been delineated [4,5], there is a need for more sensitive biomarkers to detect the malignant condition earlier and improve the ability to discriminate between a stable, asymptomatic condition and a progressive disease [6].

Telomeres are repetitive TTAGGG sequences that cap chromosome ends and prevent them from being recognized as DNA damage. With each cell division, the telomeric DNA is reduced and telomeres become progressively shorter, eventually leading to cell senescence or cell death [7]. Telomere functions depend on the minimal length of telomeric repeats and the activity of different proteins associated with them. These proteins include the shelterin complex (TRF1, TRF2, TIN2, RAP1, TPP1, POT1) that regulates telomere length (TL) and protects them against degradation [8,9], and the non-shelterin complex that comprise a set of multifunctional factors such as DNA repair proteins MRE11/NBS1/RAD50 (MNR complex) and Replication protein A1 (RPA1) that prevent telomere degradation and facilitate telomerase-based telomere elongation [10]. Maintenance of the telomere architecture involves a highly regulated network of protein-protein, protein-DNA and protein-RNA interactions; thus its impairment can result in telomere dysfunction, cellular senescence and transformation to a malignant state [11,12].

Telomerase is a ribonucleoprotein complex containing an internal RNA template (TERC) and a catalytic protein with telomere-specific reverse transcriptase activity (hTERT). By adding telomeric DNA repeat to chromosome ends, telomerase maintains TL and compensates for the continued replicative loss of telomeres. In most cases, *hTERT* expression is positively correlated with telomerase activity and with cancer initiation and progression. It is transcriptionally repressed in many normal cells and is reactivated or upregulated during immortalization [13]. In addition, other several factors are also required for the correct activity of telomerase, including dyskerin (encoded by the *DKC1* gene), which directly binds to and stabilizes TERC within the complex [14]. Point mutations in *DKC1* gene cause the X-linked form of dyskeratosis congenital (DC), a disease characterized by multiple features including abnormalities of the skin, bone marrow failure and an increased predisposition to cancer. All patients with DC display excessively short telomeres and reduced telomerase activity, suggesting that DC is manly a disease of dysfunctional telomere maintenance [15,16]. However, it is not well known how the mutations in dyskerin lead to telomere shortening. A possible explanation arises from *in vitro* experiments, in which loss of DKC1 function affect telomerase activity by reducing TERC levels and therefore leading to premature telomere shortening [17,18] that may result in chromosomal end-to-end fusions, breakage and rearrangements associated to tumor development [13,19]. Conversely, wild-type dyskerin is usually over-expressed and not mutated in sporadic cancers, although its contribution to tumorigenesis remains poorly understood.

It is already known that an imbalance in telomere-associated genes can lead to telomere dysfunction, which in turn could cause genomic instability and disease development. To date, most research on telomere in plasma cell disorders have been mainly focused on telomere shortening and telomerase activity [20–23]. Using gene expression arrays Diaz de la Guardia et al [24] identified that the upregulation of *hTERT* along with other 16 genes were involved in telomere length maintenance. Moreover, specific changes in the expression of shelterin genes and its association with clinical parameters in MM were recently described by our group [25]. However, no study has yet analyzed the interplay of *hTERT* expression and non-shelterin genes in these entities. Therefore, the aim of this study was to evaluate the expression profile of *DKC1*, *MRE11/NBS1/RAD50* and *RPA1* genes in MM and MGUS, and examine whether they are related with telomerase expression, telomere length and clinical characteristics of patients.

## Materials and Methods

### Patients

Seventy newly diagnosed patients with plasma cell disorders: 35 with MGUS and 40 with MM were analyzed. The diagnosis was based on the International Myeloma Working Group Criteria [3]. MM staging was made according to the classification proposed by Durie & Salmon [26] and the International Staging System (ISS) [27]. Clinico-pathological characteristics of all patients are summarized in Table 1. The median of follow up was 72 month (range 3–95 months). All individuals provided their written informed consent according to institutional guidelines. The study was approved by the Ethics Committee of the National Academy of Medicine.

**Table 1:** Clinical parameters of patients with plasma cell disorders.

Characteristics	MM	MGUS
**No Cases**	40	35
**Mean Age (years) (range)**	66.8 (33–87)	70.0 (41–88)
**Gender (M/F)**	18/22	15/20
**DS stages (%)**		
I	33.30	-
II	4.20	-
III	62.50	-
**ISS (%)**		
1	27.80	-
2	33.30	-
3	38.90	-
**BMI (%)**		
0- < 10	-	100
10–30	27.60	-
>30–60	31.00	-
>60	41.40	-
**Bone lesions (%)**	40.00	0
	**Mean-Range**	
**β**_**2**_ **microglobulin (μg/μL)**	0.65 (0.15–1.82)	0.29 (0.11–0.73)
**LDH (UI/L)**	211.8 (96–1265)	158.6 (94–231)
**Albumine (g/dL)**	3.26 (1.80–4.60)	3.75 (3.20–4.20)
**Calcium (mg/dL)**	9.32 (6.80–14.60)	9.18 (8.50–10.30)
**Creatinine (mg/dL)**	1.79 (0.58–11.30)	0.94 (0.46–1.82)
**Hemoglobin (g/dL)**	11.31 (6.90–15.10)	12.7 (9.90–15.10)
**M Band (g)**	3.29 (0.08–9.48)	0.62 (0.16–1.58)

MM: multiple myeloma;

MGUS: monoclonal gammopathy of undetermined significance;

M: male;

F: female;

DS: Durie & Salmon;

ISS: International Staging system;

BMI: bone marrow infiltration;

LDH: lactate dehydrogenase.

### RNA extraction, reverse transcription and quantitative PCR

Total RNA was extracted from mononuclear cells isolated from BM samples of patients, as previously reported [28]. The cDNA synthesis was performed in a final volume of 20 μl, containing 1μg of the total RNA, for 10 minutes at 95°C, for 60 minutes at 37°C and 10 minutes at 95°C to inactive the enzyme. cDNA was stored at -20°C until use. The mRNA expression of *RPA1*, *MRE11*, *NBS1*, *RAD50* and *DKC1* was determined using real-time quantitative PCR (qPCR) on a LightCycler Real-Time PCR system (Roche Diagnostics, Manneheim, Germany), based on TaqMan methodology. Primer sequences were previously described by Poncet et al [29]. Probes were specifically designed for this work (Table 2).

**Table 2:** Sequences of probes used in qPCR assays.

Gene	Probe sequence
*RPA1*	5’ ACgAgACTTCCgTCATgCCCTgTgAggAC 3’
*MRE11*	5’ CagAggTgATTgAggTAgATgAATCAgA 3’
*RAD50*	5’ ATTAgCC TCACTCATCATTCgCCTggCC 3’
*NBS1*	5’ AgAAgAgTggCTAAggCAggAA-ATggA 3’
*DKC1*	5’ CAggTgT-TCTTCgATATgAggACggCAT 3’

All PCR runs were performed in duplicate, using 4 μl of each RT reaction, 1X TaqMan master mix (Roche Diagnostics, Mannheim, Germany), 200 nM of the probe and 500 nM of each primer, in a 20 μl final volume. Analysis of *GAPDH* expression, selected as the housekeeping gene, was carried out using primers and probe described by Hu et al [30]. The thermal cycling profile for all targets started with a 95°C incubation for 10 min, followed by 45 cycles at 95°C for 15 seconds and 60°C for 1 minute. All measurements included a determination of the standards and no-template as a negative control, in which water was substituted for the cDNA. A six-point standard curve, derived from K-562 cell line cDNA, was included in each qPCR so that relative quantities of target mRNA normalized to the housekeeping gene could be determined.

### Telomere length evaluation

The TL of 33 MGUS and 34 MM patients were determined by terminal restriction fragments (TRF) assay. Briefly, genomic DNA was purified using the standard method, with proteinase K treatment and phenol/chloroform extraction, from BM samples of patients. DNA (10μg) was double digested overnight by *HinfI* and *RsaI* (Promega), fractioned on a 0.8% agarose gel and transferred to a nylon membrane by Southern blot. Hybridization and detection of the telomeric sequences were performed as previously described [28]. DNA samples from K-562 cell line were used as internal control for telomere shortening, and cord blood cells as a control for no telomere reduction. In addition, peripheral blood mononuclear cells from 30 healthy individuals (17 males and 13 females; mean age 60.31 years, range: 31–86 years) with no personal or family history of cancer, matched by sex and age were also evaluated. All of them provided their informed consent.

### Statistical evaluation

The statistical analyses were performed using the Mann-Whitney and linear regression tests where appropriate. Groupwise comparison of the distribution of clinical and laboratory variables was performed with the Student t test (for quantitative variables) and the χ^2^ or Fisher´s exact test (for categorical variables). The cutoff values for TL and *hTERT* were selected according to Receiver Operating Characteristic (ROC) analysis. Overall survival (OS) was estimated by the Kaplan-Meier method and compared with the log-rank test. For all tests, p<0.05 was considered as statistically significant.

## Results

**Fig 1:**
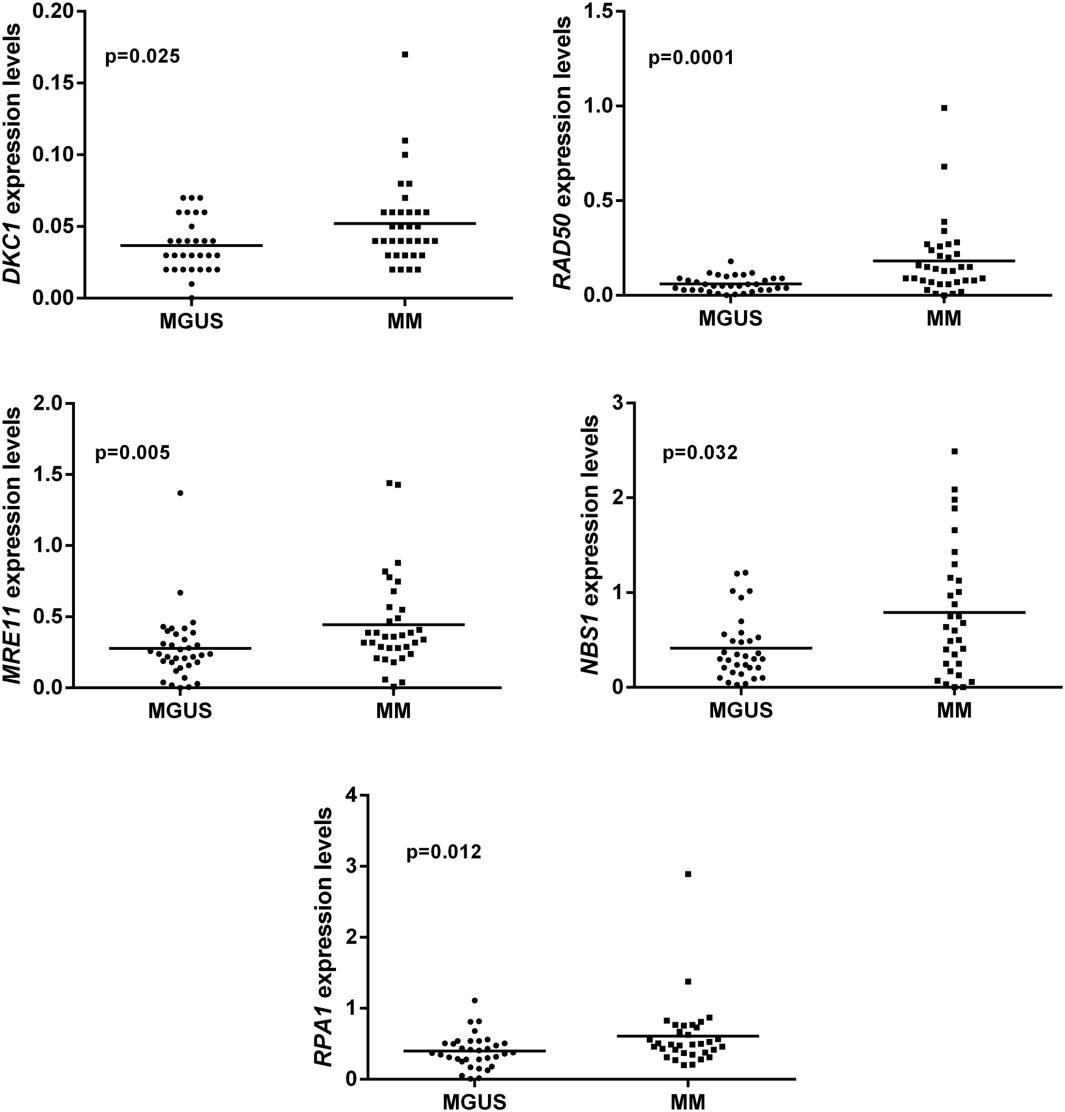
*DKC1*, *RAD50*, *MRE11*, *NBS1* and *RPA1* transcription levels in patients with MM and MGUS. A significant upregulation of the five genes in MM compared to MGUS was observed (p≤0.032).

In this study the expression profile of *DKC1*, *RPA1*, *MRE11*, *RAD50* and *NBS1* genes were examined in 35 patients with MGUS and 40 with MM. As shown in Fig 1, a significant upregulation of *DKC1* (p = 0.025), *RAD50* (p = 0.0001), *MRE11* (p = 0.005), *NBS1* (p = 0.032) and *RPA1* (p = 0.012) expression in MM compared with MGUS was detected. For both pathologies, a similar distribution of cases with mRNA levels above the mean expression for each gene was observed (MM range: 31.5%- 46%; MGUS range: 38.2%- 51.5%).

**Fig 2:**
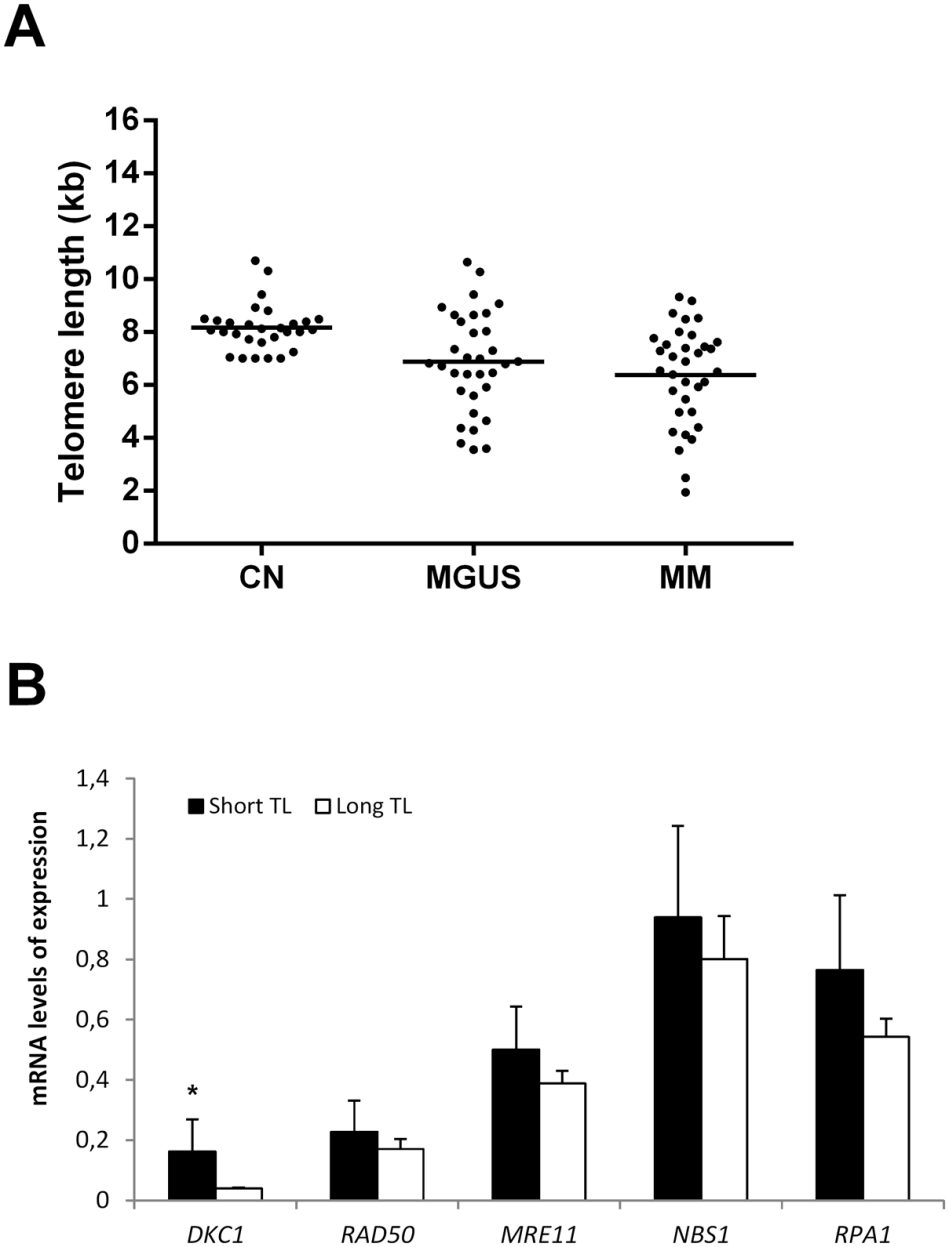
Relationship between telomere length (TL) and gene expression. A) TL was measured in 30 normal controls (NC), 33 patients with MGUS and 34 with MM. Solid lines indicated in each group represent the mean TL. Results showed significantly reduced TL in MM and MGUS patients compared with normal controls (p≤0.0005 and p≤0.002, respectively). There was no significant difference in TL between pathologies. B) Influence of TL on gene expression in MM. Patients were divided into two groups with a cutoff value of 6.15 kb, obtained by ROC curve analysis. Short TL: telomeres ≤6.15kb; Long TL: telomeres >6.15kb. *Significant differences were observed for *DKC1* expression (p = 0.026).

TL measured by TRF assay, was evaluated in 30 healthy individuals, 34 cases with MM and 33 with MGUS. Overall, patients exhibited significantly shorter telomeres than controls (p≤0.0005) (Fig 2A). The TL in healthy individuals ranged from 7 to 10.70 kb, with a mean TL of 8.16±0.17 kb. In contrast, the mean TL for MM was 6.38±0.40 kb (range: 1.94–9.33 kb) and the mean TL for MGUS was 6.84±0.35 kb (range: 3.55–10.65 kb). No significant difference in TL between both pathologies was found. Based on the TRF results, we next investigated whether there is a relationship between non-shelterin complex and TL. Both MM and MGUS patients were divided into two groups: short and long TL, with a cutoff value obtained by ROC curve analysis (6.15). The comparison of gene expression profile between groups revealed an association between short telomeres and high mRNA expression levels in both pathologies. Particularly in MM, significant differences for *DKC1* were observed (p = 0.026) (Fig 2B).

**Fig 3:**
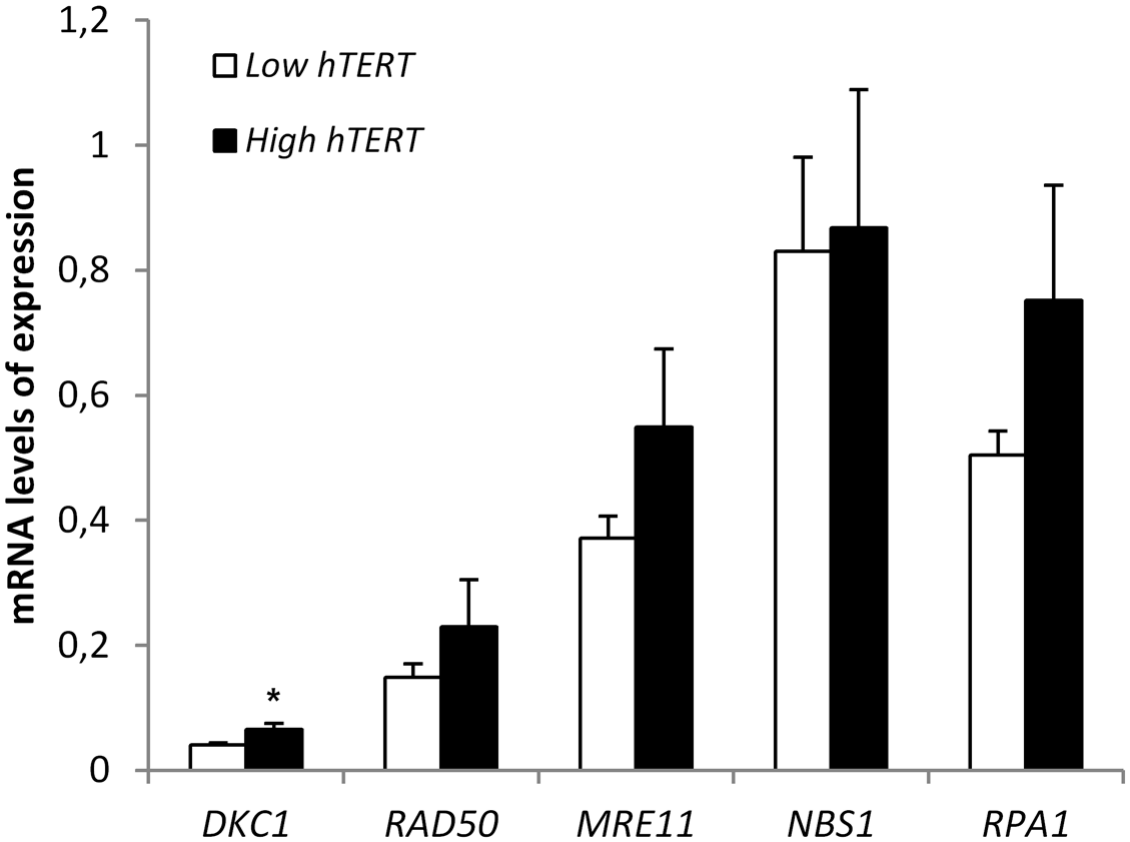
Histogram showing the expression profile of non-shelterin genes taking into account telomerase mRNA levels in MM patients. The expression cutoff value for *hTERT* was 1.05, based on ROC curve analysis. *hTERT* low: expression level ≤ 1.05; *hTERT* high: expression level > 1.05. *Significant differences were observed for *DKC1* expression (p = 0.024).

As known, DKC1 is a component of the telomerase enzymatic complex, and *RPA1* and MRN complex participate along with telomerase in telomere maintenance. In this context, we further analyzed if the transcript level of any of the non-shelterin genes evaluated in the present study was related to telomerase mRNA expression. As previously reported [25], *hTERT* mRNA expression was higher in MM (mean: 3.61±0.80) than in MGUS cases (mean: 1.52±0.38) (p = 0.029). Next, we compared non-shelterin transcript levels between patients with low or high *hTERT* expression, taking into consideration the cutoff value for *hTERT* expression (1.05) obtained by ROC curve analysis. Interestingly, both pathologies exhibited a similar profile, where patients with high *hTERT* mRNA levels were found to show higher non-shelterin genes expression (Fig 3). Significant differences were observed for *DKC1* in MM (p = 0.024), supporting the concept that DKC1 may be important to maintain telomerase activity.

**Fig 4:**
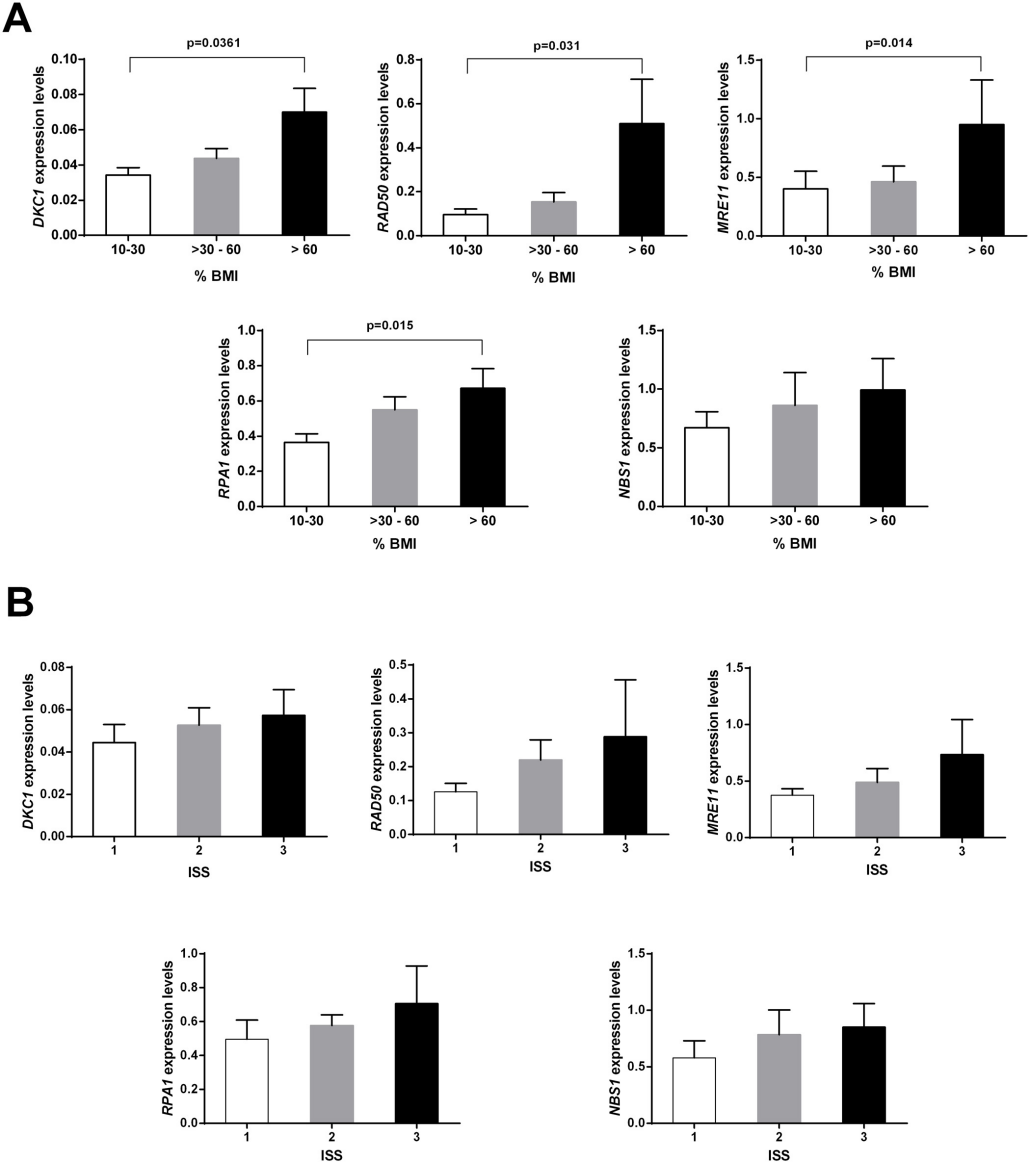
Analysis of clinico-pathological parameters in MM. A) Significant differences in the mean expression of *DKC1*, *MRE11*, *RAD50* and *RPA1* in patients with 10–30% bone marrow infiltration (BMI) with respect to those with >60% BMI (p≤0.0361). B) A tendency towards high expression of the five non-shelterin genes with advanced international staging system (ISS) stage.

Analyses of correlation between gene expression and the clinicopathological parameters were performed. High expression of *DKC1*, *RAD50*, *MRE11* and *RPA1* were positively associated with increased BM infiltration in MM (p≤0.03) (Fig 4A). In addition, a tendency towards high expression of the five non-shelterin genes with advanced ISS stage was observed (Fig 4B). No significant association between gene expression and age, gender, hemoglobin, calcium, creatinine, and β2microglobulin level was found. Finally, although no significant association among gene expression and clinical evolution were observed, patients with high *DKC1* expression showed shorter OS (54 months) than those displaying low *DKC1* transcription levels, who did not reach median survival. More studies in a larger cohort will be necessary to confirm these results.

## Discussion

In addition to telomerase and shelterin proteins, various interacting factors are involved in the regulation of telomere structure and functions. For instance, components of the shelterin complex are known to interact with DNA repair signaling pathways to control cell fate when telomeres are damaged [31]. It is well documented that the altered expression of telomerase and shelterin proteins confer malignant cells the ability to bypass senescence while promoting genomic instability [19,32]. However, limited reports have addressed the role of non-shelterin complex in telomere dysfunction and most of the investigations were performed in breast and prostate cancer [18,33]. Therefore, in this study we investigated the expression profile of a set of non-shelterin genes involved in essential processes such as replication (*RPA1*), DNA damage repair pathways (MRN genes) and stabilization of telomerase complex (*DKC1*), in a similar number of patients with MM and MGUS. Results were correlated with *hTERT* expression, TL measurements and clinical features of patients.

Overall, our results showed for the first time a significant increase in the expression of *DKC1*, *RPA1* and *MRN* genes in MM compared with MGUS, suggesting a role for the upregulation of these interacting factors in the progression of the disease. To date, the investigations evaluating the involvement of telomere-associated genes in lymphoid malignancies are limited. The literature shows studies in chronic lymphocytic leukemia with controversial results. Poncet et al [29] found increased mRNA levels of *RPA1* but downregulation of *DKC1*, *MRE11* and *RAD50* by qPCR, whereas Hoxha et al [34] observed overexpression of *MRE11*, *RAD50*, and *RPA1* by microarray assays and subsequent qPCR validation. In both reports modifications in the expression levels of these genes represented an early event in the disease. Furthermore, *DKC1* gene overexpression was observed in MM [24] and in the pre-leukemic disorder Shwachman-Diamod syndrome [35], whereas Montanaro et al [36] detected *DKC1* expression in B-cell lymphomas by immunohistochemistry. In addition, a significant *DKC1* upregulation was detected in different types of solid tumors, including hepatocellular carcinoma [37], prostate [33], colon [38], and breast [18] cancer, suggesting a critical role for this gene in carcinogenesis.

It has also been suggested that the upregulation of these genes, in particular *RPA1*, occurs as a result of the increasing need for DNA repair [39]. RPA1 is a single-stranded DNA binding protein that plays an essential role in telomere maintenance by unfolding G-quadruplex structures formed in telomeric DNA. This process facilitates lagging strand DNA replication and telomerase activity [40]. In our study, a non-significant increase of *RPA1* in cases displaying short TL and high *hTERT* mRNA levels was observed, suggesting its collaboration in the stabilization of short telomeres. Concerning the MRN complex, it is recruited to act in the first steps of DNA double strand break repair [41,42] and to work along with telomerase and its related factors in telomere maintenance [43]. Different studies showed that the MRN complex influences TL in a telomerase-dependent manner, since the reduction of any of MRN subunits resulted in telomere shortening in telomerase-positive but not telomerase-negative cells [44]. Moreover, Wu et al [45] demonstrated that MRN promotes TRF1 (shelterin protein) phosphorylation, that results in the release of TRF1 from telomeres, leading to increased access of telomerase to chromosome ends. This model is in agreement with our previous report showing that TRF1 is the only component of the shelterin complex that is downregulated in MM and MGUS [28] and with our new findings that MRN is upregulated in cases with short telomeres and high telomerase expression. Taken together, our data provide new insights into the intricate mechanisms by which telomere-associated proteins collaborate in telomere homeostasis.

Dyskerin functions in several cellular processes, such as RNA and general protein biosynthesis [46] and stabilization of the telomerase complex [47,48], as well as regulation of apoptosis [49]. In X-linked DC, DKC1 deficiency predispose to cancer development, particularly skin cancers and leukaemias, by failing to stabilize telomerase and allowing cell proliferation in the absence of functional telomeres [16,50]. Critically short telomeres can be recognized as DNA damage causing increased chromosome instability, as telomeres from different chromosomes are fused together by DNA repair proteins, and ultimately leading to malignant transformation. Strikingly, several studies have also confirmed that *DKC1* overexpression is involved in tumorigenic processes in different types of cancer. In these studies, increased *DKC1* expression was related to markers of tumour proliferation, like high TERC [18,33], MKI67 [33,37] and MYC levels [37]. Moreover, DKC1 knockdown by siRNA treatment in prostate cancer cells affected cell proliferation but also resulted in decreased cell size and spontaneous detachment, compatible with a defect in protein biosynthesis [33]. In our study, a significant *DKC1* overexpression associated with short TL and high telomerase levels was observed in MM compared with MGUS, confirming its participation in telomere elongation and suggesting a role for this gene in cancer development. Moreover, considering that to date most of the telomere-associated proteins studied in plasma cell disorders showed increased levels of expression, we also propose that the upregulation of *DKC1* in MM may occur not only to maintain telomerase activity but also to support the increased protein biosynthesis in malignant plasma cells. Similar conclusions were observed in breast [18] and prostate cancer [33].

With regard to clinical associations, we found that MM cases with increased BM infiltration showed a significant increase in *DKC1*, *RAD50*, *MRE11* and *RPA1* expression. Additionally, a tendency towards high expression of the five non-shelterin genes in patients with advanced ISS stage was observed. Although these results need to be confirmed in a larger cohort, it is noteworthy that our study provides the first evidence of non-shelterin genes associated to risk factors in MM. Similarly, in breast cancer high *MRE11* expression was associated with a more malignant behavior of the disease, lymph node metastasis, and higher recurrence rates after radiotherapy and chemotherapy [51], whereas low *DKC1* expression and activity were related to a better prognosis [18]. Also, in hepatocellular carcinoma *DKC1* overexpression was an independent risk factor for the prognosis of the disease [37]. Therefore, these results together with our previous findings showing that shelterin genes upregulation, particularly *POT1*, are associated with poor prognosis markers [25,28], provide a further contribution to understand the clinical significance of telomere-associated genes in plasma cell disorders.

To our knowledge, the present study shows for the first time an imbalance in the expression profile of non-shelterin genes in MM and MGUS, associated with short telomeres and high telomerase expression. These findings contribute to the comprehension of the role that telomere dysfunction plays in the maintenance of plasma cells immortalization/proliferation. Nevertheless, future studies are needed to explore their participation in the pathogenesis and/or progression of the disease.
